# Mechanisms of intrinsic resistance and acquired susceptibility of *Pseudomonas aeruginosa* isolated from cystic fibrosis patients to temocillin, a revived antibiotic

**DOI:** 10.1038/srep40208

**Published:** 2017-01-16

**Authors:** Hussein Chalhoub, Daniel Pletzer, Helge Weingart, Yvonne Braun, Michael M. Tunney, J. Stuart Elborn, Hector Rodriguez-Villalobos, Patrick Plésiat, Barbara C. Kahl, Olivier Denis, Mathias Winterhalter, Paul M. Tulkens, Françoise Van Bambeke

**Affiliations:** 1 Pharmacologie cellulaire et moléculaire, Louvain Drug Research Institute, Université catholique de Louvain, Brussels, Belgium; 2 Life Sciences, School of Engineering and Science, Jacobs University, Bremen, Germany; 3 CF & Airways Microbiology Research Group, Queen’s University Belfast, Belfast, UK; 4 Laboratoire de microbiologie, Cliniques Universitaires Saint-Luc, Université catholique de Louvain, Brussels, Belgium; 5 Laboratoire de bactériologie, Hôpital Jean Minjoz, Besançon, France; 6 University Hospital Münster, Münster, Germany; 7 Laboratoire de microbiologie, Hôpital Erasme, Université libre de Bruxelles, Brussels, Belgium

## Abstract

The β-lactam antibiotic temocillin (6-α-methoxy-ticarcillin) shows stability to most extended spectrum β-lactamases, but is considered inactive against *Pseudomonas aeruginosa*. Mutations in the MexAB-OprM efflux system, naturally occurring in cystic fibrosis (CF) isolates, have been previously shown to reverse this intrinsic resistance. In the present study, we measured temocillin activity in a large collection (n = 333) of *P. aeruginosa* CF isolates. 29% of the isolates had MICs ≤ 16 mg/L (proposed clinical breakpoint for temocillin). Mutations were observed in *mexA* or *mexB* in isolates for which temocillin MIC was ≤512 mg/L (nucleotide insertions or deletions, premature termination, tandem repeat, nonstop, and missense mutations). A correlation was observed between temocillin MICs and efflux rate of *N*-phenyl-1-naphthylamine (MexAB-OprM fluorescent substrate) and extracellular exopolysaccharide abundance (contributing to a mucoid phenotype). OpdK or OpdF anion-specific porins expression decreased temocillin MIC by ~1 two-fold dilution only. Contrarily to the common assumption that temocillin is inactive on *P. aeruginosa*, we show here clinically-exploitable MICs on a non-negligible proportion of CF isolates, explained by a wide diversity of mutations in *mexA* and/or *mexB*. In a broader context, this work contributes to increase our understanding of MexAB-OprM functionality and help delineating how antibiotics interact with MexA and MexB.

*Pseudomonas aeruginosa* is the most prevalent pathogen isolated in the respiratory tract of adult cystic fibrosis (CF) patients and a major cause of morbidity and mortality in this population^1^. These patients are frequently exposed to antipseudomonal antibiotics and, as a result, become colonized by multidrug-resistant strains. Since therapeutic choices narrow, clinicians are increasingly forced to look after “forgotten” antibiotics against which resistance rates could be low because they were scarcely used^2^.

Temocillin (6-α-methoxy-ticarcillin) is one of these old antibiotics recently revived, based on a renewed interest for its activity against many β-lactam-resistant *Enterobacteriaceae*^3^. Temocillin, indeed, shows remarkable stability to most β-lactamases, including AmpC-type cephalosporinases and extended-spectrum β-lactamases (ESBLs) such as TEM, SHV, and CTX-M enzymes^4^. Temocillin also obtained an orphan drug designation for the treatment of *Burkholderia cepacia* complex infection in CF patients^5^. However, as temocillin was long considered as intrinsically inactive against *P. aeruginosa*, it was not included in conventional susceptibility testing of this organism when isolated from CF patients. Previous work from our laboratory actually showed that the intrinsic resistance of *P. aeruginosa* to temocillin was due to active efflux by the constitutively-expressed MexAB-OprM efflux transporter, while the other major efflux transporters were not involved^6^. Moreover, some isolates from CF patients regained susceptibility to temocillin because of natural mutations in the proteins constituting the MexAB-OprM efflux pump^6^. MexAB-OprM belongs to the Resistance Nodulation Division superfamily of efflux transporters. It is energized by proton motive force and consists of three proteins, namely an inner membrane exporter (homotrimer of MexB), an outer membrane gated channel (homotrimer of OprM), and a periplasmic linker (6 to 13-mer of MexA), the concerted action of which allows for the extrusion of substrates from the inner membrane or the periplasmic space directly out of the bacteria (ref. 7 for review). The role of porins in the capacity of temocillin to cross the outer membrane is unknown so far. Among porins described in *P. aeruginosa*, the cation-selective channel OprD (OccD1) is involved in the entry of carbapenems (most notably, imipenem), while the anion-specific channels OpdK (OccK1) and OpdF (OccK2) are involved in the entry of carboxypenicillins like carbenicillin and of cefoxitin^8^.

In this context, the objective of this study was to better document the mechanisms of intrinsic resistance and acquired susceptibility to temocillin in *P. aeruginosa* isolated from CF patients. We exploited a large collection of 333 isolates collected from 4 CF centres in Northern Europe, which has been partly characterized for its resistance to commonly used anti-pseudomonas agents^9^. We demonstrate that a non-negligible proportion of these isolates (~15–30%) showed low and clinically-exploitable MICs to temocillin, associated with a wide variety of mutations in *mexA* or *mexB* genes. No β-lactamase hydrolysing temocillin was found in the collection. A marginal role of anion-specific porins (OpdK and OpdF) in temocillin influx was also demonstrated. Besides its immediate interest for the management of infections in CF patients, this work also brings innovative pieces of information regarding mutations affecting the transport activity of the MexAB-OprM efflux system in *P. aeruginosa*, which can refine our understanding of the mechanism of substrate recognition and transport by this pump and may also help in reviving other antibiotics that are substrates of the same transporter.

## Results

### Activity of temocillin against clinical isolates and comparison with other β-lactams

**Figure 1:**
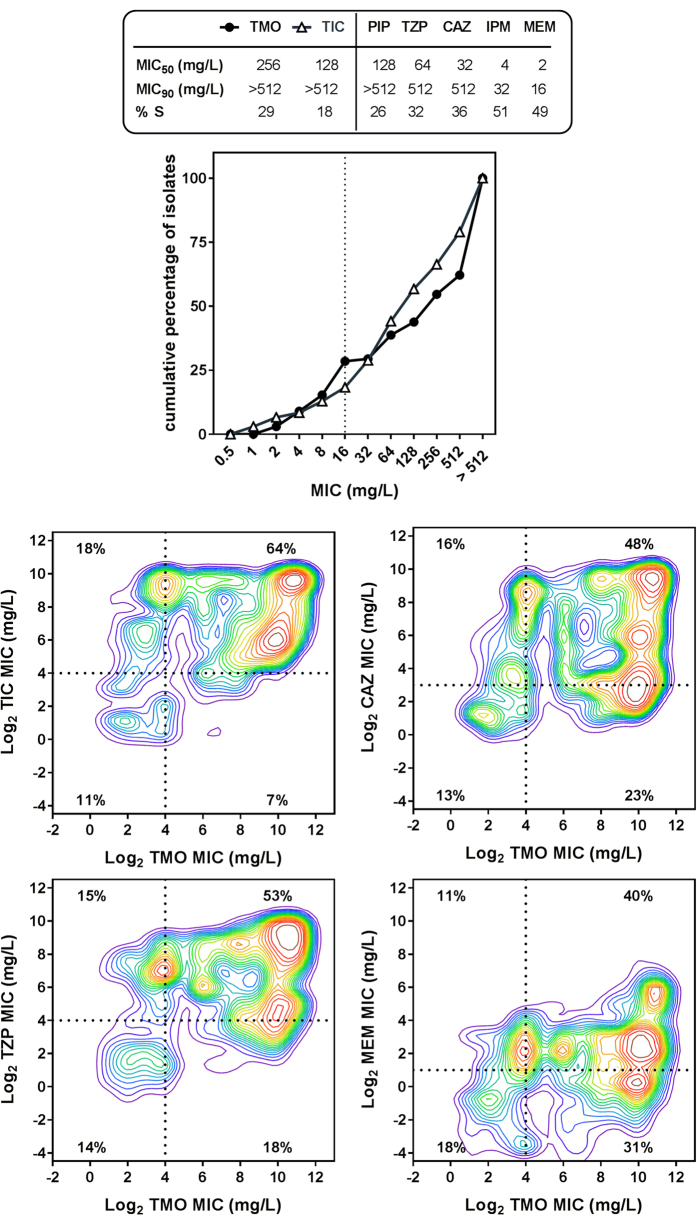
Activity of temocillin and comparators against CF isolates of *P. aeruginosa*. Panel (**a**): Cumulative MIC distribution for temocillin (TMO) compared to ticarcillin (TIC), with indication of MIC_50_, MIC_90_ and percentage of susceptibility according to the interpretive criteria of EUCAST (S, susceptible; R, resistant) for ticarcillin (S ≤ 16 mg/L; R > 16 mg/L); piperacillin (PIP, S ≤ 16 mg/L; R > 16 mg/L); piperacillin-tazobactam (TZP, S ≤ 16 mg/L; R > 16 mg/L); ceftazidime (CAZ, S ≤ 8 mg/L; R > 8 mg/L); imipenem (IPM, S ≤ 4 mg/L; R > 8 mg/L); meropenem (MEM, S ≤ 2 mg/L; R > 8 mg/L). A value of 16 mg/L (dotted line in the graph; EUCAST susceptibility breakpoint of ticarcillin) has been considered as cut-off value for temocillin (TMO), for comparison purposes. Panels (**b**–**e**): cross-resistance between TMO and other β-lactams. Correlation between MICs of TMO (abscissa) and TIC, CAZ, TZP or MEM (ordinate) for each individual isolate in the collection using quantile density contour analysis (JMP^®^ version 12.1.0). The intensity of each zone (from warm to cold colours) is indicative of the proportion of isolates (from large to small) with MICs at the corresponding coordinates. The broken lines point to the MIC value above which the isolates are considered resistant for TIC, CAZ, TZP and intermediate for MEM according to EUCAST interpretive criteria. A value of 16 mg/L has been considered for TMO. The percentage of isolates is indicated in each quadrant of the figures. MICs values are expressed as the log_2_ of their value.

Figure 1a shows the MIC distribution of temocillin compared to ticarcillin against the whole collection, with MIC_50_ and MIC_90_ of conventional antipseudomonal β-lactams illustrated in the accompanying Table. For temocillin, 15% and 29% of isolates had an MIC ≤ 8 and 16 mg/L, respectively. These values are close to those observed with ticarcillin (13% and 18%, respectively). Of interest, a similar proportion of isolates were susceptible to first-line antipseudomonal agents such as piperacillin, piperacillin/tazobactam, or ceftazidime. Carbapenems were active against a higher proportion of isolates.

Figure 1b–e shows cross-resistance between temocillin and the other β-lactams for individual isolates. While a high proportion of isolates were cross-resistant to temocillin and ticarcillin (64%), piperacillin/tazobactam (53%), ceftazidime (48%), or meropenem (40%), a small but meaningful proportion of isolates that were resistant to the comparator (ranging from 11% for meropenem to 18% for ticarcillin) remained susceptible to temocillin.

### β-lactamases screening and identification

β-lactamase production is a main mechanism of β-lactam resistance in *P. aeruginosa.* We therefore screened the collection for extended spectrum β-lactamases (ESBLs) and carbapenemases. No carbapenemases were detected using both phenotypic and genotypic methods. Moreover, detection of genes encoding CTX-M, TEM, SHV, and BEL, PER, GES, VEB, or OXA β-lactamases returned negative results in all isolates that were simultaneously resistant to CAZ and MEM (MIC > 8 mg/L).

### Influence of active efflux on temocillin activity

**Figure 2:**
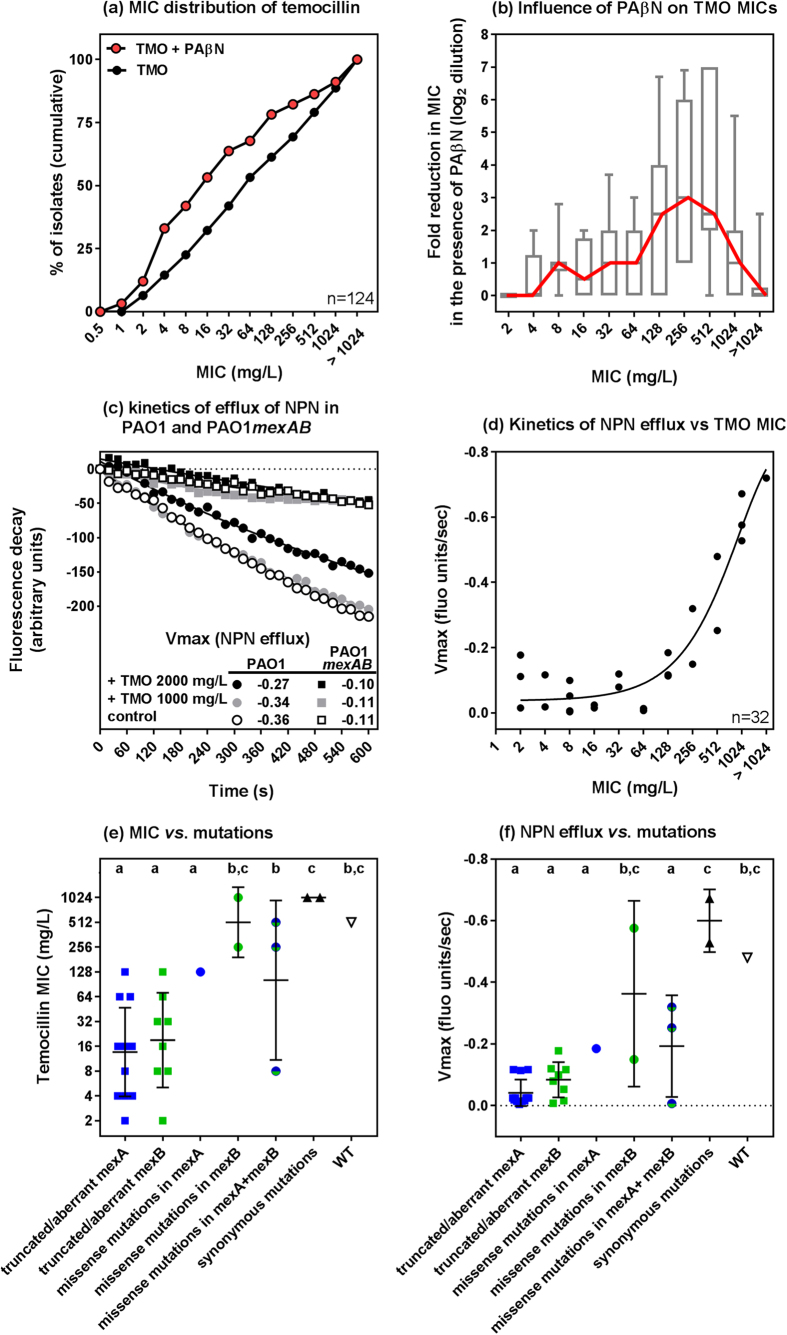
Influence of active efflux on temocillin activity. (**a**) Cumulative MIC distribution of temocillin in a subset of the collection (n = 124) selected to cover the whole range of MICs and influence of the efflux pump inhibitor PAβN (20 mg/L). (**b**) Fold reduction (log_2_ scale) in temocillin MIC in the presence of PAβN, according to temocillin MICs for the same isolates. The graph shows the box and whiskers plot with 10-90 percentiles, with the red line connecting the medians. (**c**) Kinetics of NPN efflux from PAO1 or PAO1*mexAB* in the absence (control) or presence of temocillin (TMO) at the indicated concentrations. Vmax are expressed in reduction in the fluorescence signal per second. (**d**) Kinetics of efflux of NPN as a function of temocillin MIC for the 32 isolates for which *mexA* and *mexB* were sequenced (Table 1). The ordinate is expressed as the Vmax (arbitrary fluorescence units). R^2^ for a one-phase association: 0.9043. Node splitting value for slower efflux: MIC  of 256 mg/L or lower (LogWorth statistic: 29.5157 [p < 0.001]). (**e**,**f**) MIC and rate of NPN efflux in sequenced isolates classified according to the type of mutations observed in *mexA* and *mexB* (Table 1). ‘Truncated/aberrant’ refer to deletions of more than 3 consecutive nucleotides in the sequence, nonstop mutations, insertion of minisatellites or aberrant signal peptides. ‘Missense mutations’ refer to mutations leading to the replacement of at least one amino acid in the corresponding protein. The graphs shows individual values together with means and SD. Statistical analysis (1-way ANOVA; Tuckey post-hoc test): data series with different letters are different from one another (p < 0.05).

Previous studies suggested that active efflux by MexAB-OprM plays a major role in *P. aeruginosa* resistance to temocillin^6^. Using a representative subset of isolates (n = 124) selected to cover the whole range of MICs (8–14 isolates for each MIC value), we therefore examined the influence of the broad spectrum efflux pump inhibitor Phe-Arg-β-naphthylamide (PAβN^10^) on temocillin activity. We checked that PAβN was not toxic by itself for these isolates in the conditions of the experiment. MICs were shifted towards lower values in the presence of the inhibitor (Fig. 2a), with its effect being particularly marked for isolates showing MICs ranging between 128 and 512 mg/L in the absence of PAβN (Fig. 2b). To confirm the role of MexAB-OprM-mediated efflux in resistance to temocillin, we followed in real-time the efflux of *N*-phenyl-1-naphthylamine (NPN), a preferential MexAB-OprM substrate^11^, using temocillin as a competitor and comparing PAO1 to its MexAB-OprM deletion mutant PAO1*mexAB* (Fig. 2c). NPN efflux was much slower in the deletion mutant than in the parent strain. Temocillin was able to decrease the rate of efflux of NPN in PAO1 but had no effect in the MexAB-OprM deletion mutant even when using a temocillin concentration much higher than its MIC (2 mg/L). These data strongly suggest that temocillin is a preferential substrate of MexAB-OprM. The rate of efflux of NPN was therefore evaluated in 32 isolates (29 clinical isolates plus PAO1, PT629, PAO1*mexAB*) harbouring increasing temocillin MICs (Fig. 2d). NPN efflux was significantly slower for isolates with a temocillin MIC < 512 mg/L and increased in proportion to temocillin MIC.

### Mutations in *mexA* and *mexB* in relation with temocillin MIC and efflux pump activity

MexA and MexB, but not OprM, determine the β-lactam specificity of the MexAB-OprM efflux system^12^. We therefore sequenced *mexA* and *mexB* genes in the 32 isolates for which NPN efflux was evaluated. Figure 2e,f illustrates the relationship between the type of mutations evidenced and the MIC of temocillin or the rate of efflux of NPN. Isolates harboring deletions in *mexA* or *mexB* or aberrant sequences (deletion of ≥3 consecutive nucleotides, nonsense mutation, nonstop mutation^13^, aberrant signal peptide, minisatellite repeat) had the slowest rate of efflux of NPN and temocillin MICs ranging between 2 and 128 mg/L. Isolates with missense mutations globally showed a slower efflux than PAO1 and temocillin MICs of 128 or 256 mg/L. Synonymous mutations did not impact NPN efflux or temocillin MICs. In addition, most of the isolates showed a high frequency of synonymous mutations and codon degeneracy especially at the third position. Most of them were conserved between different isolates originating from different countries.

Table 1 summarizes these results and shows in parallel for each isolate the Vmax for NPN efflux, together with (i) the MICs of temocillin in control conditions or in the presence of the efflux pump inhibitor PAβN or of the OpdK porin substrate vanillate, and (ii) the relative amount of exopolysaccharides. The constructed molecular graphics of the corresponding proteins are illustrated in Table S2 (see also Fig. S1 for a view of the wild-type proteins, with specification of their subdomains).

Tables S2b–e specifically focus on sequenced isogenic isolates collected either from the same patient or from different patients originating either from the same or from different countries. Table S2b illustrates isolates collected in the UK and belonging to the Liverpool epidemic strain (LES)^9^. Isolates from five different patients with an MIC of 16 mg/L shared the same synonymous mutations and also the same nonsense mutation in *mexA* leading to the production of a 119 amino acid protein. Of note, two other LES isolates with an MIC of 32 mg/L shared the same synonymous mutations in *mexA* and *mexB* genes as those described for the first 5 LES isolates, but harboured different deletions in their *mexB* gene, leading to proteins of 30 and 672 amino acids, respectively. Table S2c illustrates 2 pairs of isolates harbouring the same mutations although collected from different patients in the same country. Thus, in the isogenic isolates 191-4 and 207^9^ with a temocillin MIC of 64 mg/L, MexA was truncated to 27 amino acids. Likewise, in isogenic isolates W024 and W049 with a MIC of 4 mg/L, MexA was truncated to 69 residues due to the loss of 1 nucleotide leading to a premature stop codon. Interestingly also, isogenic isolates can be found in different countries (Table S2d). These three isolates belonging to the same multidrug-resistant clonal complex^9^ and originating from the UK (AG3), Belgium (128) and Germany (129-6) shared the same synonymous mutations. Only AG3 had a low MIC to temocillin (8 mg/L), associated with a MexA protein truncated in its MP domain (372 amino acids left). The two other isolates had MICs of 1024 mg/L, with one conservative missense mutation (L376V) found in the transmembrane domain TM3 of the MexB protein of isolate 128. Finally, Table S2e illustrates three other clonal isolates differing in their susceptibility to temocillin. They come from two different patients in Germany and share (i) a radical missense mutation (G616T) in the *mexA* part encoding the third domain of MexA interacting with the distal domain of MexB, (ii) a conservative missense mutation (A556G), and (iii) a deletion of nucleotides 1947 and 1948 in *mexB*. In addition, isolate 135-1, with a temocillin MIC of 8 mg/L, showed an insertion in *mexB* (G1261_C1262insG) leading to the production of an aberrant and truncated MexB protein. In contrast, isolates 208-3 and 208-2, with temocilllin MIC of 256 and 512 mg/L, differed only by the position of an additional 1 nucleotide deletion (in position 1854 or 1889, respectively) in the *mexB* gene part encoding the pore (PC1) subdomain, leading to the replacement of 30 by 29 amino acids, and of 19 by 18 amino acids, respectively. This difference could explain the 1 two-fold dilution difference in temocillin MIC between these two isolates.

Despite the evidenced truncations or mutations in MexA and MexB, most of the clinical isolates showed nevertheless higher temocillin MICs than the reference strain PAO1*mexAB* lacking MexA and MexB proteins (see Fig. 2e). This strongly suggests that other mechanisms of resistance independent of the functionality of MexAB-OprM are present.

### Culture content in extracellular polymeric saccharides with β-1, 4 and β-1, 3 linkages

*P. aeruginosa* isolated from CF patients often harbour a mucoid phenotype, related to the overproduction of extracellular polysaccharides, among which alginate contributes to the chronicity of infections. Alginate production has been shown to impede the activity of β-lactams, including ticarcillin^14^,^15^.

**Figure 3:**
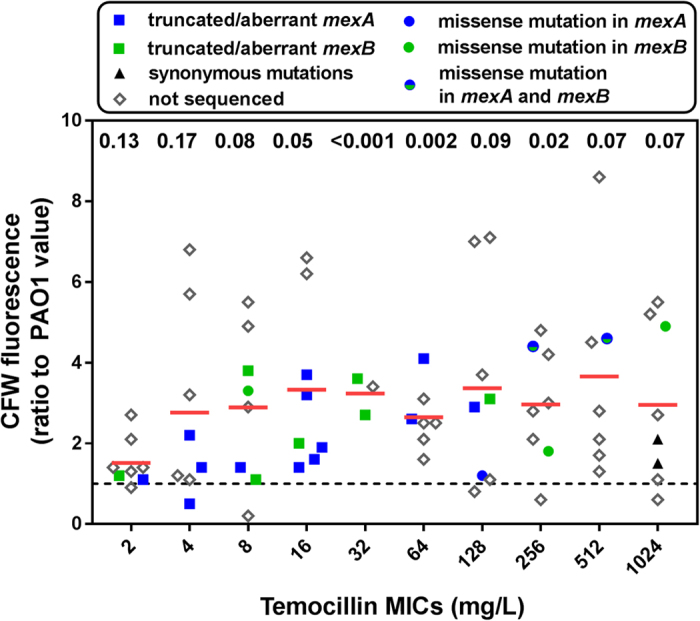
Evaluation of exopolysaccharide abundance in cultures of clinical isolates as compared to the reference strain PAO1 as a function of temocillin MICs. Calcofluor white (CFW) fluorescence was measured for 73 clinical isolates, including those for which *mexA* and *mexB* were sequenced (Table 1). Data expressed as a ratio to the value measured for the non-mucoid reference strain PAO1 (highlighted by the dotted line), the MIC of which is 512 mg/L). The red horizontal lines show the mean of values for 7–8 isolates for each MIC (3 only for 32 mg/L [no more isolates with this MIC value in the whole collection]). Statistical analysis: figures above each bar represents the p value (multiple t-test) versus PAO1. Node splitting value for significant increase in CFW fluorescence signal *vs*. PAO1: MIC = 4 mg/L or higher LogWorth statistic: 1.6343 [p 0.02].

We therefore compared exopolysaccharide abundance in cultures of 73 clinical isolates (included those which had been sequenced) *vs.* PAO1 by measuring calcofluor white binding to polysaccharides (Fig. 3). A high variability in fluorescence values was observed among isolates, but a global statistical analysis showed that the increase in fluorescence signal was significant for isolates with a temocillin MIC ≥ 4 mg/L. Notably, among sequenced isolates with truncated or aberrant MexA or MexB, the fluorescence signal was at least 2.6-fold higher than in PAO1 for those for which temocillin MICs are between 32 to 128 mg/L.

### Influence of porins on temocillin activity

β-lactams are known to cross the outer membrane of Gram-negative bacteria via porins.

While OprD (OccD1) has been well characterized as facilitating the entry of carbapenems (mainly imipenem^16^) into *P. aeruginosa*, other members of the OprD family like OpdK (OccK1, archetype of the OccK subfamily^17^) and OpdF (OccK2) are involved in the transport of the carboxypenicillin carbenicillin and of the anionic cephalosporin cefoxitin^8^. As temocillin is also a carboxypenicillin, we examined whether these anion-selective porins were involved in temocillin transport into *P. aeruginosa.* OprD was studied in parallel. Other members of the OprD family of porins were not investigated in the present work.

MICs of temocillin, cefoxitin, imipenem, and meropenem were measured in transformants of a porin-deficient *E. coli* strain expressing OpdK, OpdF, or OprD (Table 2; carbenicillin could not be tested due to the presence of a β*-*lactamase-encoding gene on the transforming plasmid). Consistent with previous studies^8^, OprD expression increased the activity of carbapenems but not that of the other drugs. Conversely, the expression of OpdK, and to a slightly lesser extent, of OpdF, increased the activity of temocillin and cefoxitin; this effect was best seen when the antibiotics were combined with PAβN.

**Figure 4:**
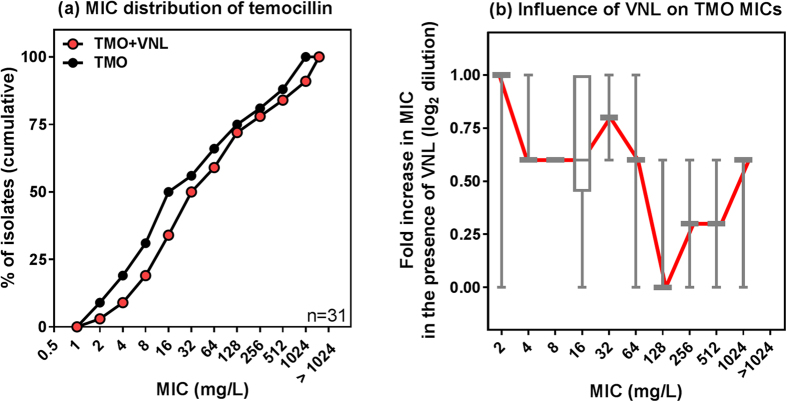
Influence of porins on temocillin activity. (**a**) Cumulative MIC distribution of temocillin in a subset of the collection (n = 31) selected to cover the whole range of MICs and influence of the OpdK competitor vanillate (VNL 2 mM). (**b**) Fold reduction (log_2_ scale) in temocillin MIC in the presence of VNL, according to temocillin MICs for the same isolates. The graph shows the box and whiskers plot with 10–90 percentiles, with the red line connecting the medians.

Subsequently, MICs were measured in *P. aeruginosa* mutants of each of these three porins (Table 3). MICs for carbapenems were markedly (3–4 log_2_ dilutions) increased by the loss of OprD while those of temocillin and carbenicillin were not affected. Conversely, deletion of OpdK or OpdF modestly (<1 log_2_ dilution) increased the MIC of carboxypenicillins. This effect was amplified in the presence of vanillate (substrate for OpdK^17^,^18^) in the OpdF mutant for carboxypenicillins, suggesting that these two porins cooperate for the import of these antibiotics. Likewise, L-Arginine (substrate for OprD^19^) reduced carbapenem activity in the OpdK or OpdF mutants. The effect of vanillate on temocillin activity against clinical isolates is illustrated in Fig. 4a (MIC distribution) and in Table 1 (individual isolates). This effect was modest, but best seen in isolates with temocillin MICs ≤ 64 mg/L, in which efflux was deficient (Fig. 4b).

## Discussion

This study is the first to describe the activity of temocillin in a large collection of *P. aeruginosa* collected from CF patients, and to determine the mechanisms that can lead to a phenotype of ‘acquired susceptibility’ to this antibiotic, which is uncommon in this pathogen^3^. Our results showed that a low but significant proportion of the isolates have MICs lower than the current temocillin BSAC (British Society for Antimicrobial Chemotherapy) susceptibility breakpoint for systemic infections, and to the current EUCAST breakpoint for the parent molecule ticarcillin, as well as to the breakpoint proposed for high dosing regimens of temocillin based on pharmacokinetic/pharmaocodynamic considerations^20^. Even if far from being the majority, these susceptible isolates cannot be ignored, as 30 to 60% of them are resistant to conventional antipseudomonal β-lactams, including carbapenems, making temocillin one of the last viable therapeutic option.

We confirm that altered active efflux mediated by MexAB-OprM is the main driver of restored susceptibility to temocillin. Non-functional efflux, as evidenced using NPN as a tracer, was observed in isolates with temocillin MICs < 512 mg/L, and these also harbour mutations in *mexA* and/or *mexB* leading to protein alterations. Among the sequenced isolates, we could evidence a whole panel of mutations that may lead to loss of functionality, many of which had not been reported in our earlier study of temocillin-susceptible CF isolates^6^, neither in other studies investigating the effect of *mexA/B* mutations on the pump activity^21^,^22^,^23^.

The most susceptible isolates show major deletions in *mexA* or *mexB* leading to the production of truncated or aberrant proteins. Over the 8–512 mg/L range of MICs, *mexA* mutations are found in the regions encoding the α-helical hairpin and/or of the second, third, and membrane-proximal (MP) domains, which interact with MexB and/or OprM proteins^24^, possibly affecting the linkage with MexB or OprM, and therefore the correct assembly of the tripartite efflux system^22^. *mexB* mutations are located either in the regions building the pore domain (PC1, PC2, PN1, and PN2) implicated in drug recognition and extrusion^25^, or in those encoding the periplasmic tip of the docking subdomains (DN and DC) involved in the interaction with OprM^25^. Only one isolate (3319) showed an additional amino acid substitution outside these domains, namely in the cytoplasmic C-terminal domain of MexB^25^. Deletions in transmembrane helices TM8-12 were also identified in a few isolates. TM10 has been shown to mediate proton translocation (with the TM4) in the inner membrane domains^26^. Of interest, we noticed different mutations and hence, different susceptibility to temocillin, among clonal isolates, suggesting a high adaptability of each of them to its own environment.

Of note, most of the isolates also showed a high frequency of synonymous mutations and codon degeneracy especially at the third position. Most of these mutations were conserved among several isolates from different countries. Synonymous substitutions are often considered as silent mutations, but they can impact gene transcription and mRNA transport or translation, which could alter protein folding and function^27^. Codon degeneracy is described as highly frequent in *P. aeruginosa*^28^.

Intriguingly, restoration of temocillin activity was only partial in some isolates that showed major deletions in *mexA* or *mexB.* Since we did not investigate the impact of each evidenced mutation on temocillin MIC in a PAO1 background, we cannot exclude that this is due to the expression of other, still undescribed mechanisms of resistance, or to an overexpression of other efflux systems that may compensate for the loss of MexAB-OprM activity, as previously described in CF isolates^29^. However, we previously showed that these systems only play a marginal role in temocillin extrusion^6^. Therefore, we suggest a possible phenotypic resistance related to an overproduction of extracellular polymeric substances, including alginate, in isolates with temocillin MICs ≥ 4 mg/L. Alginate has been shown to impair the diffusion, and therefore the activity, of ticarcillin, the parent compound of temocillin^14^,^15^. We show here that alginate production is systematically low in isolates with a temocillin MIC of 2 mg/L, *i.e*. a value corresponding to that measured for the *mexAB* deletion mutant of the non-mucoid reference PAO1, while it is high in clinical isolates with inactive efflux but temocillin MICs of 32–128 mg/L. Of note, we were able to exclude or minimize the role of two known mechanisms of resistance to β-lactams, namely the expression of β-lactamase(s) and reduced expression of porins. Thus, no β-lactamase among the few ones capable of hydrolysing temocillin (VIM, NDM, IMP, OXA-48^30^) was genotypically detected in our isolates. For porins, while OprD alterations have been previously evidenced in this collection^31^, we show here that, in contrast to carbapenems, temocillin does not require this porin to enter bacteria, but rather uses the anion-specific porins, OpdK and OpdF, the role of which seems redundant. Temocillin indeed shares a methoxy moiety with cefoxitine and vanillate, two well-established substrates of these porins. This moiety has been described for vanillate as interacting with the L3-L7 loops forming the internal constriction of OpdK^18^. Yet, the importance of these porins in temocillin uptake seems rather marginal since their inhibition increases temocillin MIC of 1 two-fold dilution only. For this reason, we did not specifically look for mutations or reduced expression of these porins in the collection. We cannot exclude that other porins expressed by *P. aeruginosa*^32^ that were not investigated here also play a role in temocillin uptake. We acknowledge this is a limitation of this work.

Considering all data, and despite remaining uncertainty regarding the reason for variable susceptibility to temocillin in mutated isolates, our work brings three major pieces of scientific progress. Firstly, from a clinical perspective, we demonstrate a potential use for temocillin in the management of *P. aeruginosa* infections in CF patients. Although limited to a small proportion of isolates, this is important as temocillin susceptibility was observed for some isolates that were resistant to other β-lactams, either by production of β-lactamases or by alteration of the OprD porin, as well as in isolates belonging to clones described as multidrug-resistant. Thus our data call for introducing temocillin in routine susceptibility testing for *P. aeruginosa* collected from CF patients. Moreover, temocillin may offer the advantage of acting at the same time on *Burkholderia* spp^5^ that can co-infect CF patients.

Second, in a context of drug design, we observed a large diversity of mutations in *mexA* and *mexB*, opening perspectives for studying their role in substrate recognition and/or transport activity. The mutants described here could also be used to study the efflux of other antibiotics and to help delineating how these interact with specific domains of MexA and MexB. Third, from an ecological perspective, our data reinforce the concept that a functional MexAB-OprM pump is not needed for *P. aeruginosa* survival in the CF lung^29^ and illustrates the high adaptability of this bacteria to its environment.

## Materials and Methods

### *P. aeruginosa* isolates

A total of 333 isolates were randomly collected from 155 patients in four CF centres from different countries (*Hôpital des enfants malades Reine Fabiola/Erasme*; Belgium, n = 88; *Hôpital Jean Minjoz*, Besançon, France; n = 80; University Hospital of Münster, n = 66, Germany; Queen’s University of Belfast, UK, n = 99) during routine periodic visits (Table S1). PAO1, mutants thereof overexpressing or deleted for MexAB-OprM (Table 1), and PA14 were used as control strains. The molecular typing of part of this collection has been previously performed by pulse field gel electrophoresis, focusing on isolates collected simultaneously from the same patients but differing in their susceptibility profile^9^. It was completed here for selected sequenced isolates following the same procedure.

### Antibiotics

The following antibiotics were obtained as microbiological standard from Sigma-Aldrich, St Louis, MO (sodium salts, potency in brackets): ticarcillin (TIC; 85.25%), piperacillin (PIP; 94.20%), carbenicillin (CAR; 89.16%), and cefoxitin (FOX; potency, 95.11%). The remaining antibiotics were obtained as powder for parenteral use from their corresponding manufacturers (potency in brackets): temocillin (TMO, 78.12%) as Negaban^®^ from Eumedica (Manage, Belgium), piperacillin-tazobactam (TZP; 97.00%) as Tazocin^®^ from Wyeth (Louvain-La-Neuve, Belgium), ceftazidime (CAZ, 88.20%) as Glazidim^®^ from Glaxo-SmithKline (Genval, Belgium), imipenem (IPM, 45.60% [due to the presence of cilastatin in the powder]) as Tienam^®^ from MSD (Brussels, Belgium), and meropenem (MEM, 74.00%) as Meronem^®^ from AstraZeneca (Brussels, Belgium).

### Susceptibility testing

MICs were determined by broth microdilution following the guidelines from the Clinical and Laboratory Standards Institute and using *P. aeruginosa* ATCC27853 as a quality control. Susceptibility was assessed according to the interpretive criteria of the European Committee on Antimicrobial Susceptibility Testing (EUCAST) or using a cut-off value of 16 mg/L for temocillin (no EUCAST breakpoint) by analogy with the current susceptibility breakpoint of its parent compound ticarcillin. For specific isolates, MICs were also measured in the presence of 20 mg/L Phe-Arg-β-naphthylamide dihydrochloride (PAβN, broad-spectrum efflux pump inhibitor^10^; Sigma-Aldrich) and of 1 mM MgSO_4_ (to prevent the outer membrane permeabilization caused by PAβN^33^) or in the presence of substrates of *P. aeruginosa* porins, namely vanillate (VNL, OpdK substrate^18^), or L-arginine (L-Arg, OprD substrate^19^), both obtained from Sigma-Aldrich.

### β-lactamases screening and identification

Isolates showing MICs > 8 mg/L for both ceftazidime and meropenem (n = 53) were screened for the presence of genes encoding acquired metallo-β-lactamases (VIM, IMP, NDM), carbapenemases (OXA-48, KPC), or widespread extended-spectrum β-lactamases (ESBLs) by PCR, as previously described^9^. The expression of carbapenemase(s) was also screened using a phenotypic method described as the Carbapenemase Nordmann-Poirel (Carba NP) test^34^.

### Sequencing of *mexA* and *mexB*

*mexA and mexB* genes were amplified by PCR using the following primers: *mexA-F* (5′-GCGAGGCTTTCGGACGTTTA-3′); *mexA-R* (5′-GGCAGACTGAGGATCGACA-3′), *mexB1-F* (5′-CAAGGGGATTCGTAATGTC-3′); mexB1-R (5′-GTGAACATCCAGATCATCC-3′), *mexB2-F* (5′-CGGATGTTCCTTTCCACCAC-3′); *mexB2-R* (5′-GACAGAACGACAGCGGCTA-3′). Annealing temperatures for each pair of primers and amplicon sizes were as follows: *mexA*, 60 °C, 1375 bp; *mexB1*, 61 °C, 1684 bp; *mexB2*, 67 °C, 1638 bp. Sanger sequencing was performed in forward and reverse directions at GATC Biotech (Konstanz, Germany) using the same primers. PAO1 was used as a reference. Molecular graphics for MexA and MexB proteins were rendered using Visual Molecular Dynamics program (VMD^35^), based on Protein Data Bank (PDB) files 2V4D (MexA^24^), and 2V50 (MexB^25^).

### Direct measurement of efflux activity

Efflux pump functionality was assayed by following the kinetics of efflux of *N*-phenyl-1-naphthylamine (NPN; Sigma-Aldrich), a MexAB-OprM preferential substrate^11^, which is fluorescent when incorporated in bacterial membranes. As previously described^36^, bacteria were treated with carbonyl cyanide *m*-chlorophenylhydrazone (CCCP, 10 μM; Sigma-Aldrich) and then loaded with 20 μM NPN. Efflux was initiated by energizing bacteria with 50 mM D-glucose (Sigma-Aldrich). Decay in fluorescence signal was followed over time using a Spectramax^®^ multiplate reader (Molecular devices, Sunnyvale, CA). Maximal velocity (Vmax) was calculated by the SoftMax^®^ Pro Microplate Data Acquisition and Analysis Software (version 6.2). PAO1*mexAB* (Δ*mexAB*-mutant) was used as negative control.

### Cloning and overexpression of porins

Genes encoding different porins *i.e.* OprD (OccD1), OpdK (OccK1), and OpdF (OccK2) were cloned in a heterologous system of *Escherichia coli*, which was lacking the main native porins OmpC and OmpF (*E.coli* K-12 W3110:Δ*ompF*Δ*ompC*)^37^. The genes *opdK* and *opdF* were PCR amplified from PAO1 (with HindIII and NsiI restriction site overhangs) and further cloned into pGOmpF (a vector with the signal sequence of *phoE* [phosphoporin protein E] attached to the *ompF* gene^38^) via HindIII/PstI, thereby replacing only *ompF*. This resulting vectors (pG-OpdK or pG-OpdF) had thus *opdK* and *opdF* attached to the *phoE* signal sequence and was further under the control of the *lac* promoter. The expression of *opdK or opdF* was inducible by 0.5 mM IPTG. The pB22-OprD construct was a kind gift from Prof. Bert van den Berg, Newcastle, UK. In this *E. coli* expression vector, the *oprD* gene with the signal sequence of the *E. coli* outer membrane protein YtfM is under the control of the P_BAD_ promoter inducible by 0.5% (w/v) arabinose. MICs were determined in the presence of inducer.

### Single-porin *P. aeruginosa* mutants

Insertion mutants for the same 3 porins were obtained from the PA14 transposon insertion mutant library from the Harvard Medical School, Boston, MA^39^. This transposon library has been previously characterized and sequenced in order to identify insertion sites^40^; all transformants were resistant to gentamicin, confirming the expression of the gentamicin-resistance gene present on the insertion cassette.

### Quantification of extracellular polymeric saccharides (EPS)

EPS were quantified using calcofluor white (BD*™* Diagnostics, Sparks Glencoe, MD), a fluorophore which binds to β-(1, 4) and β-(1, 3) polysaccharides^41^, including alginate (co-polymer of O-acetylated β-(1, 4) D-mannuronic acid and L-guluronic acid), a major constituent of slime produced by mucoid *P. aeruginosa*. Bacteria were cultured in 96-well plates as for MIC determinations (starting inoculum, 10^6^ CFU/mL; 24 h incubation), after which bacterial density was evaluated by measuring OD_620nm_. The content of the wells was transferred to Eppendorf tubes and calcofluor white at a final concentration of 30 mg/L was added. The tubes were incubated for 30 minutes at room temperature and subsequently centrifuged at 20,800* g*. Pellets were washed with phosphate buffered saline (PBS pH 7.4), resuspended in the same buffer, and transferred in black 96-well plates to measure fluorescence in a Spectramax^®^ multiplate reader (λ_exc_ 370 nm; λ_em_ 440 nm). Fluorescence values were normalized *vs.* the OD_620nm_ of the cultures, with the value measured for PAO1 set to 1 as a reference.

### Statistical analyses

Statistical analysis was performed using JMP^®^ version 12.1.0, SAS Institute Inc., Cary, NC, USA or GraphPad Prism version 7.01 (GraphPad software Inc., San Diego, CA).

### Ethics

The protocol for this study has been examined by the ethical committee of the *Université catholique de Louvain*, who determined that it did not fall under the scope of the law on human experimentation as (i) all isolates were collected during routine sampling and assembled retrospectively, and (ii) all patients’ data were anonymized.

## Additional Information

**How to cite this article:** Chalhoub, H. *et al*. Mechanisms of intrinsic resistance and acquired susceptibility of *Pseudomonas aeruginosa* isolated from cystic fibrosis patients to temocillin, a revived antibiotic. *Sci. Rep.* **7**, 40208; doi: 10.1038/srep40208 (2017).

**Publisher's note:** Springer Nature remains neutral with regard to jurisdictional claims in published maps and institutional affiliations.

## Supplementary Material

Supplementary Material

## Figures and Tables

**Table 1:** Efflux characteristics, MIC and relative polysaccharide content for reference strains versus CF clinical isolates of *P. aeruginosa.*

Reference strains and clinical isolates (patient’s identification code; country; date of collection)	Description (phenotype; length of MexA/B protein; AA deletions and affected MexA/B domains)	Efflux characteristics			MIC (mg/L)			EPS (β-1, 4/β-1, 3 glycosidic bonds)
		*mexA* alterations	*mexB* alterations	Vmax (units/s)	TMO	TMO +PAβN	TMO+VNL	
PAO1	Wild type MexA (383 AA) MexB (1046 AA)	—	—	−0.480	512	128	768	1
PT629^a^	PAO1 MexAB-OprM overproducer	—	—	−0.720	1024	256	1536	
PAO1*mexAB*^a^	PAO1 *ΔmexAB*	—	—	−0.112	2	2	2	
3724 (AL1; France; 14/12/2006)	-MexA (383 AA).-Truncated MexB (878 AA): **S879_Q1046del** deletion of 168 AA affecting 5 TMs (TM8-TM12).	-Uncommon^b^ synonymous mutations: C546G-Prevalent^b^ synonymous mutations: A345G G993A	-Uncommon synonymous mutations: C486T-Prevalent synonymous mutations: A495G C1308T C1692T T2280C T2730C G3117A**-Nonsense mutation: C2636A**	−0.178	2	2	4	1.2
144 (PCF79; Belgium; 20/09/2010)	-Truncated MexA (124 AA): **A125_G383del** deletion of 259 AA affecting the α-hairpin, second, third and MP domains.-MexB (1046 AA)	-Prevalent synonymous mutation: G993A**-Nonsense mutation, Δ 13 nt (367–379)**	Prevalent synonymous mutations: A495G T2280C T2730C G3117A	−0.016	2	2	4	1.1
W024 (DM; UK; 2009)	-Truncated MexA (69 AA): **V70_G383del** deletion of 314 AA in α-hairpin, second, third and MP domains.-MexB (1046 AA)	**Nonsense mutation, Δ 1 nt (164)**	No mutations	−0.117	4	2	6	1.4
W049 (DB; UK; 2009)	-Isogenic to W024-Truncated MexA (69 AA): **V70_G383del** deletion of 314 AA in α-hairpin, second, third and MP domains.-MexB (1046 AA)	**Nonsense mutation: Δ1 nt (164)**	No mutations	−0.117	4	2	6	2.2
143-1 (143; Germany; 12/07/2012)	-Nonstop mutation in *mexA* (**stalled ribosome**, proteolysis; recycled ribosome)^13^-MexB (1046AA)	-Prevalent synonymous mutation: G993A-**Nonstop mutation: Δ 2 nt (837–838)**	-Uncommon synonymous mutations: C2412T-Prevalent synonymous mutations:A495G T2226C T2280C T2730C G3117A	−0.019	4	4	8	0.5
AG3 (JP; UK; 10/05/2006)	-Shortened MexA (372 AA): **G337_E347del** deletion of 11 AA in the MP domain.-MexB (1046AA)	-Prevalent synonymous mutations: A345G G993A-**Deletion: Δ 33 nt (1011–1043)**	Prevalent synonymous mutations: C474T C492T A495G T2280C T2730C G3117A	−0.004	8	4	12	1.4
129 (RCF62; Belgium; 09/09/2010)	-MexA (383 AA).-MexB (1045 AA): **N254del** deletion of asparagine 254 in the DN subdomain and missense mutation **(T557N)** in TM7.	-Uncommon synonymous mutations: C633T G702A C780T-Prevalent synonymous mutations: T333C A345G G447A A639G T655C T729C C732T A738G C789G G792A T1002C T1095C	-Uncommon synonymous mutations: C909T G1362A-Prevalent synonymous mutations: A495G T2730C G3117A-**Conservative missense mutation: C1670A**-**Deletion: Δ3 nt (760–762)**	−0.053	8	2	12	1.1
135-1 (135; Germany; 10/07/2012)	-MexA (383 AA): **A206S** missense mutation (third domain).-**MexB truncated** (719 AA): **M720_Q1046del** deletion of 327 AA in DC, PN1, PC2 and TM8-TM12 and **300 AA substitutions (I186V; M421_719)** in TM5-7, DN, PC1, and PC2.	-Uncommon synonymous mutations: G438T G558C-Prevalent synonymous mutations: T333C A345G G447A A639G T655C T729C C732T A738G C789G G792A C951G T1002C T1095C-**Radical missense mutation: G616T**	-Uncommon synonymous mutations: G348A C1101T C1389A-Prevalent synonymous mutations: C315T A351G A495G C498G G591A C600G C537T A1938G T2067C A2079G T2280C T2730C-**Conservative missense mutation: A556G**-**Insertion and deletion leading to premature stop codon: G1261_C1262insG + Δ 2 nt (1947–1948)**	−0.100	8	8	12	3.8
126 (VIF68; Belgium; 09/09/2010)	-SCV isolate.-MexA (383 AA): aberrant signal peptide **(S19L)**.-MexB (1046 AA): missense mutations **(I186V, Q773P)** in DN and DC subdomains at the OprM-docking domain of MexB	-Uncommon synonymous mutations: C582T C912T-Prevalent synonymous mutations: T333C A345G G447A T655C T729C C789G G792A C951G T1002C T1095C-**Radical missense mutation: C56T**	-Uncommon synonymous mutations:C1314A G2004A C2014T-Prevalent synonymous mutations: C315T A351G A495G C498G C537T G591A C600G A804C G1587T T1749C A1938G T2067C A2079G T2226C T2280C T2730C T2826C A2892G C2955T-**Conservative missense mutation: A556G**-**Radical missense mutation: A2318C**	−0.007	8	2	12	3.3
279 (RRM59; Belgium; 28/10/2010)	-MexA (383 AA).-Aberrant MexB (1027 AA): **K151_T169del** deletion of 19 amino acids in the periplasmic pore domain (subdomain PN2)	No mutations	-Prevalent synonymous mutations: A351G C1308T T2280C T2730C G3117A-**Deletions: Δ57 nt (455–511)**	−0.016	16	4	24	2
BM1 (AD; UK; 13/09/2006)	- LES isolate.-Truncated MexA (119 AA): **Y120_G383del** deletion of 264 AA in α-hairpin, second, third and MP domains.-MexB (1046 AA)	-Prevalent synonymous mutation: A345G-**Nonsense mutation: C360G**	-Uncommon synonymous mutation: T2015G-Prevalent synonymous mutations: A495G, C1692T, T2280C, T2730C, G3117A	−0.025	16	16	16	3.7
AJ3 (ML; UK; 22/05/2006)	- LES isolate.-Truncated MexA (119 AA): **Y120_G383del** deletion of 264 AA in α-hairpin, second, third and MP domains.-MexB (1046 AA)	-Prevalent synonymous mutation: A345G-**Nonsense mutation: C360G**	-Uncommon synonymous mutation: T2015G-Prevalent synonymous mutations: A495G, C1692T, T2280C, T2730C, G3117A	−0.025	16	16	32	1.4
CF15 (CT; UK; 2007)	-LES isolate.-Truncated MexA (119AA): **Y120_G383del** deletion of 264 AA in α-hairpin, second, third and MP domains.-MexB (1046 AA)	-Prevalent synonymous mutation: A345G-**Nonsense mutation: C360G**	-Uncommon synonymous mutation: T2015G-Prevalent synonymous mutations: A495G, C1692T, T2280C, T2730C, G3117A	−0.025	16	16	24	3.2
CF53 (DP; UK; 2007)	-LES isolate.-Truncated MexA (119 AA): **Y120_G383del** deletion of 264 AA in α-hairpin, second, third and MP domains).-MexB (1046 AA)	-Prevalent synonymous mutation: A345G-**Nonsense mutation: C360G**	-Uncommon synonymous mutation: T2015G-Prevalent synonymous mutations: A495G, C1692T, T2280C, T2730C, G3117A	−0.025	16	16	32	1.6
CF19 (LS; UK; 2007)	-LES isolate.-Truncated MexA (119 AA): **Y120_G383del** deletion of 264 AA in α-hairpin, second, third and MP domains).-MexB (1046 AA)	-Prevalent synonymous mutation: A345G-**Nonsense mutation: C360G**	-Uncommon synonymous mutation: T2015G-Prevalent synonymous mutations: A495G, C1692T, T2280C, T2730C, G3117A	−0.025	16	16	24	1.9
CF16 (RC; UK; 2007)	-LES isolate.-MexA (383 AA).-Truncated MexB (30 AA): **P31_Q1046del** only TM1 is encoded.	Prevalent synonymous mutation: A345G	-Uncommon synonymous mutation: T2015G-Prevalent synonymous mutations: A495G, C1692T, T2280C, T2730C, G3117A-**Nonsense mutation: Δ 154 nt (85–239)**	−0.120	32	8	48	2.7
BV1 (DC; UK; 11/10/2006)	-LES isolate.-MexA (383 AA).-Truncated MexB (672 AA): **E673_Q1046del** deletion of 374 AA in DC, PN1, PC2 and TM8-TM12, and 154 AA substitutions (F519_G672) in TM6-7, PC1 subdomains.	Prevalent synonymous mutation: A345G	-Uncommon synonymous mutation: T2015G-Prevalent synonymous mutations: A495G, C1692T, T2280C, T2730C, G3117A-**Nonsense mutation: Δ 8 nt (1555–1562)**	−0.080	32	8	64	3.6
191-4 (191; Germany; 03/09/2012)	-Truncated MexA (27 AA): **E28_G383del** deletion of 356 AA.-MexB (1046 AA).	-Prevalent synonymous mutation: G993A-**Nonsense mutation: G82T**	Prevalent synonymous mutations: A495G T2280C T2730C G3117A	−0.014	64	32	128	4.1
207 (207; Germany; 2012)	-Truncated MexA (27 AA): **E28_G383del** deletion of 356 AA.-MexB (1046 AA).	-Prevalent synonymous mutation: G993A-**Nonsense mutation: G82T**	Prevalent synonymous mutations: A495G T2280C T2730C G3117A	−0.014	64	32	64	2.6
109 (ENM88; Belgium; 03/09/2010)	-MexA (383 AA).-15 base pairs (5 AA residues PAIAP [P36_P40]) **minisatellite repeat** in MexB (1051 AA), toxic or malfunctioning proteins.	-Uncommon synonymous mutation: G117A C468T-Prevalent synonymous mutations: T333C A345G G447A T729C T1002C	-Uncommon synonymous mutations: G1452A C1920T-Prevalent synonymous mutations: C315T A351G A495G T642C A804C C1308T T1749C A1938G T2067C A2079G T2226C T2280C**-15 nt minisatellite repeat: 118–132**^c^	−0.008	64	toxic	64	2.5
618 (FJ1; France; 01/01/1996)	-Truncated MexA (297 AA): **V298_G383del**, deletion of 86 AA in MP domain.-MexB (1046 AA)	-Prevalent synonymous mutation: A345G-**Nonsense mutation: Δ1 nt (C869)**	Prevalent synonymous mutations: A495G C1308T C1851T T2067C T2226C T2280C T2730C T2826C A2892G C2955T G3117A	−0.114	128	toxic	128	2.9
3179 (MP1; France; 03/02/2004)	-SCV isolate.-MexA (383 AA).-Truncated MexB (719 AA): **M720_Q1046del** deletion of 327 AA in TM8-TM12, DC and PN1/PC2 subdomains.	Prevalent synonymous mutation: G993A	-Prevalent synonymous mutations: A495G C1308T C1851T T2067C T2226C T2280C T2730C T2826C A2892G C2955T G3117A-**Nonsense mutation: Δ1 nt (G2147)**	−0.117	128	128	192	3.1
180-3 (180; Germany; 30/07/2012)	-MexA (383 AA): **W332R** missense mutation in MP domain;-MexB (1046 AA)	-Prevalent synonymous mutations: A345G T729C T1002C-**Radical missense mutation: T994C**	-Uncommon synonymous mutation: G1608A-Prevalent synonymous mutations: C492T A495G T2280C T2730C G3117A	−0.185	128	32	128	1.2
3319 (BV1; France; 03/08/2004)	-MexA (383 AA);-MexB (1046 AA): **Y182C, A707S, I963V, S1041E and V1042A**, missense mutations in PC2, DN, TM11, and cytoplasmic C-terminal domain.	-Uncommon synonymous mutations: G51T C147G C291T G399A C423T G426A C591T C651T C687T C705T C777A-Prevalent synonymous mutations: T333C A345G G447A T655C T729C C951G T1002C	-Uncommon synonymous mutations: C129T G165A T423G T519C G1080A C1152T T1332C C1383T C1476G T1563C C1581T C1614T A1713G G1748C T1758G G1836C C1980T G2001T C2070A C2134T A2139G C2148T C2223T G2319A T2457C G2469C C2604A T2907C T3033C-Prevalent synonymous mutations: A351G A495G C498G C537T T642C A804C G1587T T1749C T2067C T2226C T2280C T2730C A2892G C2955T-**Radical missense mutation: G2119T**-**Conservative missense mutations: A545G; A2887G; G3120T T3121G C3122A C3123G T3125C**	−0.150	256	32	384	1.8
208-3 (208; Germany; 09/08/12)	-Isogenic to 135-1.-MexA (383 AA): missense mutation **A206S** (third domain).-MexB (1045 AA): missense mutation **I186V** (DN subdomain), and **30 AA replaced by 29 new AA** (R620_R650 in PC1 subdomain).	-Uncommon synonymous mutations: G438T G558C-Prevalent synonymous mutations: T333C A345G G447A A639G T655C T729C C732T A738G C789G G792A C951G T1002C T1095C-**Radical missense mutation: G616T**	-Uncommon synonymous mutations: G348A C1101T-Prevalent synonymous mutations: C315T A351G A495G C498G C537T G591A C600G A1938G T2067C A2079G T2280C T2730C-**Conservative missense mutation: A556G**-**Deletions: Δ 1 nt (T1854) + Δ 2 nt (1947–1948)**	−0.320	256	256	256	4.4
208-2 (208; Germany; 09/08/12)	-Isogenic to 135-1.-MexA (383 AA): missense mutation **A206S** (third domain).-MexB (1045 AA): missense mutation **I186V** (DN subdomain), and **19 AA replaced by 18 new AA** (L631_R650 in PC1 subdomain)	-Uncommon synonymous mutations: G438T G558C-Prevalent synonymous mutations: T333C A345G G447A A639G T655C T729C C732T A738G C789G G792A C951G T1002C T1095C-**Radical missense mutation: G616T**	-Uncommon synonymous mutations: G348A C1101T-Prevalent synonymous mutations: C315T A351G A495G C498G C537T G591A C600G A1938G T2067C A2079G T2280C T2730C-**Conservative missense mutation: A556G**-**Deletions: Δ 1 nt (T1889) + Δ 2 nt (1947–1948)**	−0.253	512	256	512	4.6
128 (DAF69; Belgium; 09/10/2010)	-Isogenic to AG3.-MexA (383 AA).-MexB (1046 AA): **L376V**, missense mutation in TM3.	Prevalent synonymous mutations: A345G G993A	-Prevalent synonymous mutations: C474T C492T A495G T2280C T2730C G3117A-**Conservative missense mutation: C1126G**	−0.576	1024	1024	1024	4.9
129-6 (129; Germany; 11/07/2012)	-Isogenic to AG3.-Synonymous mutations in MexA (383 AA) and MexB (1046 AA)	Prevalent synonymous mutations: A345G G993A	Prevalent synonymous mutations: C474T C492T A495G T2280C T2730C G3117A	−0.672	1024	256	1536	2.1
4289 (JV1; France; 13/08/2007)	-Synonymous mutations in MexA (383 AA) and MexB (1046 AA)	Prevalent synonymous mutations: G993A	Prevalent synonymous mutations: A495G T2280C T2730C G3117A	−0.528	1024	512	1536	1.5

^a^See ref. 6 for references.

^b^Uncommon synonymous mutations: rare mutations found in isolates belonging to only one clone or one single patient; prevalent synonymous mutations: identical mutations found in different isolates belonging to different clones.

^c^The presence of minisatellites could be consecutive to bacterial exposure to genotoxic compounds^42^,^43^; the resulting proteins are malfunctioning.

The table shows (a) the mutations detected in *mexA* and *mexB* and the changes in the corresponding proteins, the Vmax for NPN efflux (see Fig. 2, panels c-d), (b) temocillin MICs in control conditions, or in the presence of 20 mg/L PAβN as efflux pump inhibitor and 2 mM vanillate (VNL) as OpdK substrate, and (c) the culture content in exopolysaccharides relative to PAO1 (see Fig. 3). Abbreviations used: TM, transmembrane α-helix; MP, membrane-proximal domain; PN1/PN2 (periplasmic, N*-*terminal), PC1/PC2 (periplasmic, C*-*terminal), DN (docking, N-terminal) and DC (docking, C-terminal) are six periplasmic subdomains that build the pore and docking domains of MexB, respectively (see Fig. S1 for more details); nt, nucleotide; AA, amino acids; SCV, small colony variant; LES, Liverpool Epidemic Strain; EPS, extracellular polymeric saccharides. Molecular graphics for *mexA* and *mexB* mutants are represented in the supplementary data (Table S2a–e) together with the position of the different protein domains.

**Table 2:** Influence of porins on antibiotic activity in *E. coli* transformants expressing *oprD/Occd1, opdK/occK1*, or *opdF/occK2.*

Strains	Antibiotic MIC (mg/L)^a^					
	TMO		FOX		IPM	MEM
	control	+PAβN^b^	control	+PAβN^b^		
W3110 Δ*ompF/C* + pBAD-Empty	40	18	80	20	1	1.5
W3110 Δ*ompF/C* + pBAD-*oprD*	40	18	80	20	**0.225**	**0.225**
W3110 Δ*ompF/C* + pG-Empty	40	18	80	20	1	1.5
W3110 Δ*ompF/C* + pG-*opdK* clone 1^c^	**20**	**9**	**56**	**16**	1	1.5
W3110 Δ*ompF/C* + pG-*opdK* clone 2^c^	**20**	**9**	**56**	**16**	1	1.5
W3110 Δ*ompF/C* + pG-*opdF* clone 1^c^	**22**	**14**	**60**	**18**	1	1.5
W3110 Δ*ompF/C* + pG-*opdF* clone 2^c^	**22**	**14**	**60**	**18**	1	1.5

^a^MICs (mg/L) of temocillin (TMO), cefoxitin (FOX), imipenem (IPM), and meropenem (MEM) were determined in the presence of Isopropyl β-D-1-thiogalactopyranoside (IPTG) 0.5 mM or arabinose 0.5% as gene inducers. Values in bold highlight increased activity *vs*. the corresponding control (no porin expressed).

^b^20 mg/L PAβN added as an inhibitor of *E.coli* AcrAB-TolC efflux pump.

**Table 3:** Influence of porins on antibiotic activity in *P. aeruginosa* PA14 and its single disruptants for the *oprD, opdK*, or *opdF* genes.

Strain	Antibiotic MIC (mg/L)^a^											
	TMO			CAR			MEM			IPM		
	control	+VNL	+L-Arg	control	+VNL	+L-Arg	control	+VNL	+L-Arg	control	+VNL	+L-Arg
PA14	256	**384**	256	48	**64**	48	0.37	0.37	**1**	1	1	**2**
PA14::*oprD*	256	**384**	256	48	**64**	48	*3*	*3*	*3*	*4*	*4*	*4*
PA14::*opdK*^b^	*384*	384	*384*	*64*	64	*64*	0.37	0.37	**1**	1	1	**2**
PA14::*opdF*^b^	*384*	***786***	*384*	*64*	***128***	*64*	0.37	0.37	**1**	1	1	**2**

^a^MICs (mg/L) of temocillin (TMO), carbenicillin (CAR), imipenem (IPM), and meropenem (MEM) were determined in the presence of 2 mM vanillate (VNL) or 10 mM L-Arg as competitor substrates for OpdK and OprD, respectively. Values in bold highlight decreased activity *vs*. the corresponding control (no porin substrate added); values in italics highlight decreased activity vs the wild-type strain PA14.

^b^Two different clones transformed with pG-OpdK or pG-OpdF tested.
