# Supplementary material for: Mechanisms of intrinsic resistance and acquired susceptibility of Pseudomonas aeruginosa isolated from cystic fibrosis patients to temocillin, a revived antibiotic
Source: Sci Rep. 2017 Jan 16;7:40208. doi: 10.1038/srep40208 (PMC5238406; doi:10.1038/srep40208)
Supplement: Supplementary Material [file srep40208-s1.doc]

**SUPPLEMENTARY MATERIAL**

**Mechanisms of intrinsic resistance and acquired susceptibility of *Pseudomonas aeruginosa* isolated from cystic fibrosis patients** **to temocillin, a revived antibiotic**

Hussein Chalhoub^1^, Daniel Pletzer^2,#^, Helge Weingart^2^, Yvonne Braun^2^, Michael M. Tunney^3^, J. Stuart Elborn^3^, Hector Rodriguez*-*Villalobos^4^*,* Patrick Plésiat^5^, Barbara C. Kahl^6^, Olivier Denis^7^, Mathias Winterhalter^2^, Paul M. Tulkens^1^, and Françoise Van Bambeke^1,*^

^1^ Pharmacologie cellulaire et moléculaire, Louvain Drug Research Institute, Université catholique de Louvain, Brussels, Belgium.

^2^ Life Sciences, School of Engineering and Science, Jacobs University, Bremen, Germany.

^3^ CF & Airways Microbiology Research Group, Queen's University Belfast, Belfast, UK.

^4^ Laboratoire de microbiologie, Cliniques Universitaires Saint-Luc, Université catholique de Louvain, Brussels, Belgium.

^5^ Laboratoire de bactériologie, Hôpital Jean Minjoz, Besançon, France.

^6^ University Hospital Münster, Münster, Germany.

^7^ Laboratoire de microbiologie, Hôpital Erasme, Université libre de Bruxelles, Brussels, Belgium.

^#^ current affiliation : Centre for Microbial Diseases and Immunity Research, Department of Microbiology and Immunology, University of British Columbia, Vancouver, Canada

**Fig. S1. Molecular representations of MexA and MexB monomers in PAO1, with indication of their respective domains (**based on references^1-3^).

Many significant advances examining the molecular interactions between MexA, MexB and OprM were done recently.

The MexA rod domain (constructed by the long α-helical hairpin of twisted coiled-coil) interacts with OprM. The second domain (adjacent to the rod α-helical domain; globular; cluster of 8 short β-sheets) interacts with OprM and MexB. The third domain (globular; 7 short β-sheets and one short α-helix) and membrane proximal domain (MP, β-roll) are located distal to the α-helical rod. They interact with the distal domain of MexB.

The multidrug transporter MexB has 12 transmembrane segments (TMs) inserted in the inner membrane. The periplasmic pore consists of 4 subdomains (PC1, PC2 [periplasmic, C-terminal], PN1, PN2 [periplasmic, N-terminal]), which are directly involved in the export of substrates. The docking domain (DC [docking, C-terminal] and DN [docking, N-terminal] subdomains) is at the periplasmic tip of MexB and interacts with the outer membrane protein (OprM). The DN subdomain forms a long protruding loop that inserts into the docking domain of the neighbouring MexB subunit.

| Structure of MexA monomer (PAO1) | Structure of MexB monomer (PAO1) |
| --- | --- |
| The 4 domains are represented with AA residues numbered according to the *P. aeruginosa* MexA sequence: α-hairpin (red), second (yellow), third (violet), and membrane proximal MP domains (cyan). | *P. aeruginosa* MexB docking domains: DC (violet), DN (yellow); pore domains: PC1/ PC2 (Dark/clear grey), PN1/ PN2 (cyan, lime); transmembrane segments: TMs are numbered from 1 to 12. |
| 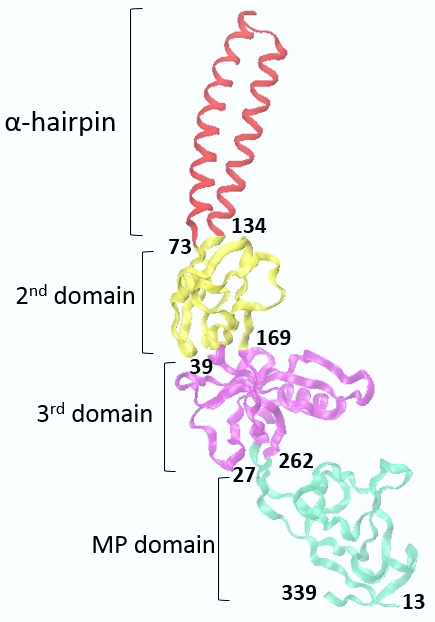 | 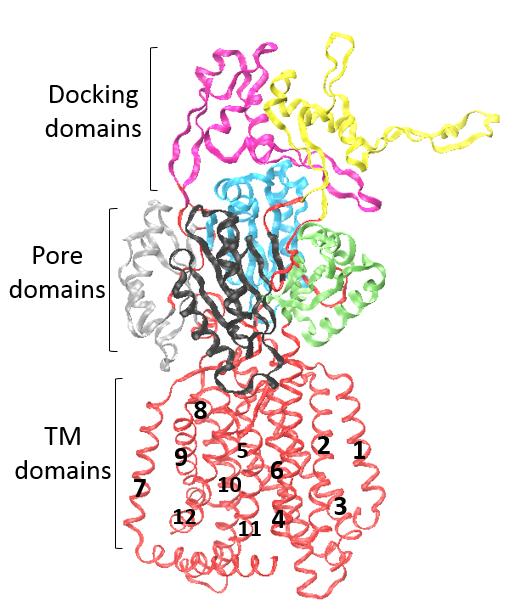 |

**References to Fig. S1:**

1. Akama H., *et al.* Crystal structure of the membrane fusion protein, MexA, of the multidrug transporter in Pseudomonas aeruginosa. *J Biol Chem.* **279,** 25939-25942 (2004).
2. Symmons M. F., Bokma E., Koronakis E., Hughes C., Koronakis V. The assembled structure of a complete tripartite bacterial multidrug efflux pump. *Proc. Natl. Acad. Sci. U S A* 106, 7173-7178 (2009).
3. Sennhauser G., Bukowska M. A., Briand C., Grutter M. G. Crystal structure of the multidrug exporter MexB from Pseudomonas aeruginosa. *J. Mol. Biol.* **389**, 134-145 (2009)

**Table S1: General description of the collection from 1996-2012**

| **Country** | **Number of isolates** | **Number of patients** | **Year of sampling** |
| --- | --- | --- | --- |
| **United Kingdom** | 99 | 46 | 2006-2009 |
| **Belgium** | 88 | 37 | 2010 |
| **France** | 80 | 36 | 1996-2012 |
| **Germany** | 66 | 36 | 2012 |
| **Total** | 333 | 155 |  |

**Table S2.** Molecular representations for MexA and MexB proteins in clinical isolates of *P. aeruginosa* isolated from cystic fibrosis patients, as compared to the wild-type PAO1.

Color code for tables S2a-e: blue: deleted residues; green: nonsynonymous substitutions of amino acids; black: tandem repeated sequence of amino acids; red: encoded parts of MexA and MexB proteins.

**Table S2a: non-clonal isolates**

| **Isolates** | **TMO**  **MIC**  **(mg/L)** | **Protein length**  **(AA)** | | **Encoded MexA** | | **Encoded MexB** | |
| --- | --- | --- | --- | --- | --- | --- | --- |
|  |  | **MexA** | **MexB** | **Side view** | **Top view** | **Side view** | **Top view** |
| **PAO1**  **wild type** | 512 | 383  (13-mer) | 1046  (trimer) | 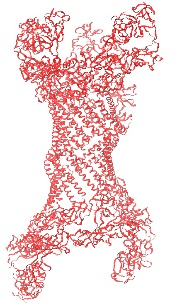 | 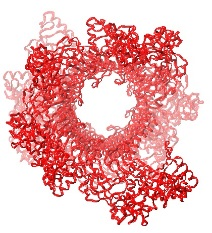 | 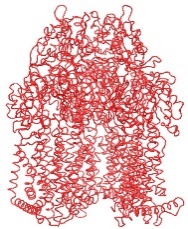 | 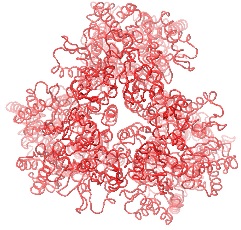 |
| **3724** | 2 | 383 | 878 | Synonymous mutations | | 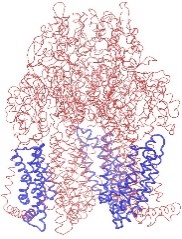 | 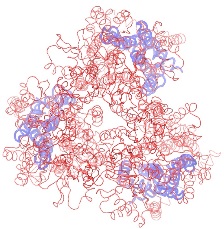 |
| **144** | 2 | 124 | 1046 | 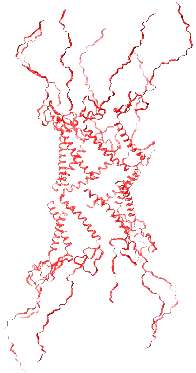 | 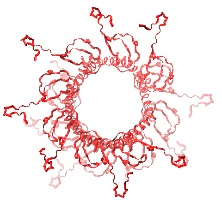 | Synonymous mutations | |
| **143-1** | 4 | 0 | 1046 | Nonstop mutation in *mexA* (proteolysis; recycled ribosome) | | Synonymous mutations | |
| **129** | 8 | 383 | 1045 | Synonymous mutations | | 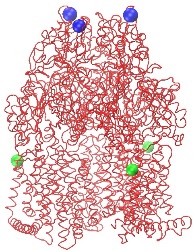 | 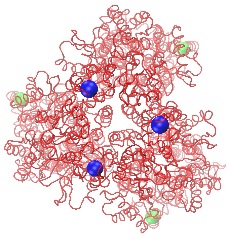 |
| **126** | 8 | 383 | 1046 | Radical missense mutation (S19L) in the signal sequence  of MexA | | 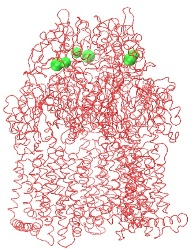 | 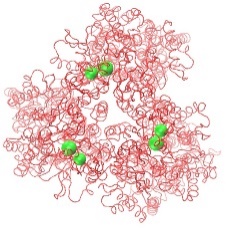 |
| **279** | 16 | 383 | 1027 | No mutations | | 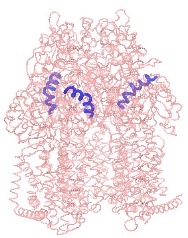 | 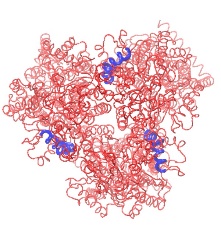 |
| **109** | 64 | 383 | 1051 | Synonymous mutations | | 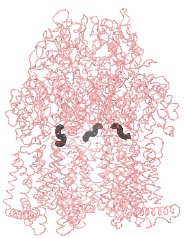 | 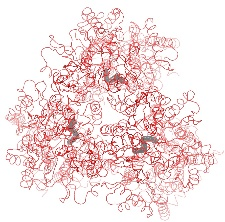 |
| **618** | 128 | 297 | 1046 | 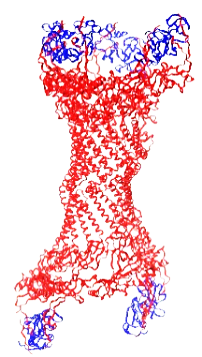 | 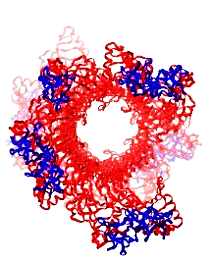 | Synonymous mutations | |
| **3179** | 128 | 383 | 719 | Synonymous mutations | | 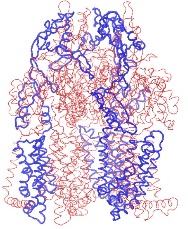 | 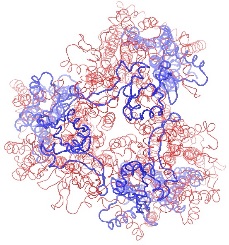 |
| **180-3** | 128 | 383 | 1046 | 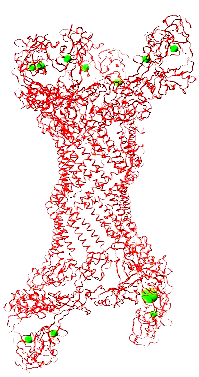 | 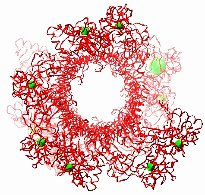 | Synonymous mutations | |
| **3319** | 256 | 383 | 1046 | Synonymous mutations | | 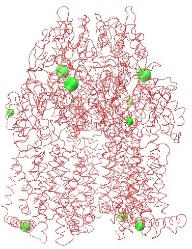 | 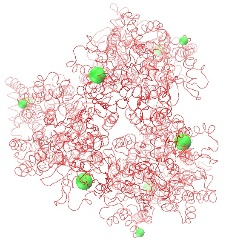 |
| **4289** | 1024 | 383 | 1046 | Synonymous mutations | | Synonymous mutations | |

**Table S2b: LES clonal isolates**

| **Isolates** | **Patient ID, country and date of collection** | **TMO**  **MIC**  **(mg/L)** | **Protein length**  **(AA)** | | **Encoded MexA** | | **Encoded MexB** | | |
| --- | --- | --- | --- | --- | --- | --- | --- | --- | --- |
|  |  |  | **MexA** | **MexB** | **Side view** | **Top view** | **Side view** | **Top view** | |
| **BM1, AJ3, CF15, CF53, CF19** | AD, UK (Sept. 2006), ML, UK (May 2006), CT, UK (2007) DP, UK (2007) LS, UK (2007) | 16 | 119 | 1046 | 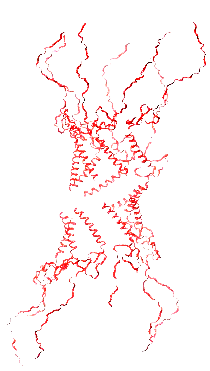 | 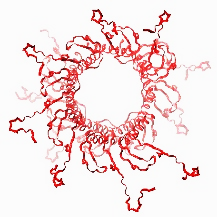 | Synonymous mutations | | |
| **CF16** | RC, UK (2007) | 32 | 383 | 30 | Synonymous mutations | | **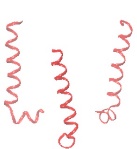** | | **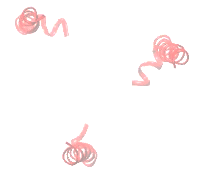** |
| **BV1** | DC, UK (Oct. 2006) | 32 | 383 | 672 | Synonymous mutations | | 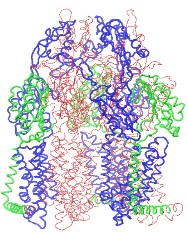 | | 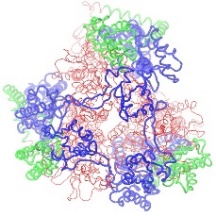 |

**Table S2c: isogenic isolates from 2 other pairs of patients in the same country, sharing the same mutations**

| **Isolates** | **Patient ID, country and date of collection** | **TMO**  **MIC**  **(mg/L)** | **Protein length**  **(AA)** | | **Encoded MexA** | | **Encoded MexB** | |
| --- | --- | --- | --- | --- | --- | --- | --- | --- |
|  |  |  | **MexA** | **MexB** | **Side view** | **Top view** | **Side view** | **Top view** |
| **191-4, 207** | 191, Germany (2012), 207, Germany (2012) | 64 | 27 | 1046 | Nonsense mutation: G82T | | Synonymous mutations | |
| **W024, W049** | DM, UK (2009),  DB, UK (2009) | 4 | 69 | 1046 | 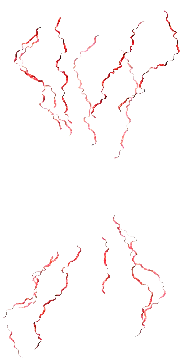 | 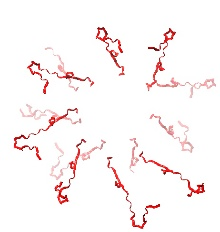 | No mutations | |

**Table S2d: isogenic isolates from three different patients originating from different coutries**

| **Isolates** | **Patient ID, country and date of collection** | **TMO**  **MIC**  **(mg/L)** | **Protein length**  **(AA)** | | **Encoded MexA** | | | **Encoded MexB** | |
| --- | --- | --- | --- | --- | --- | --- | --- | --- | --- |
|  |  |  | **MexA** | **MexB** | **Side view** | | **Top view** | **Side view** | **Top view** |
| **AG3** | JP, UK (May 2006) | 8 | 372 | 1046 | 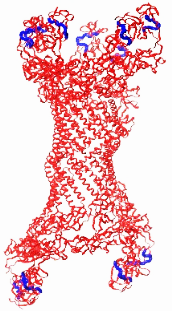 | **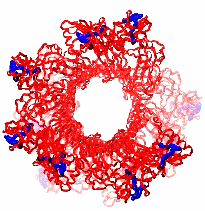** | | Synonymous mutations | |
| **128** | DAF69, Belgium (Oct. 2010) | 1024 | 383 | 1046 | Synonymous mutations | | | 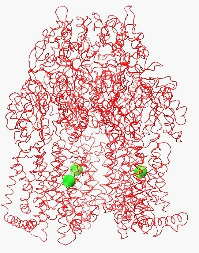 | 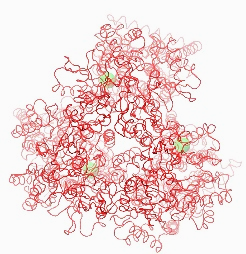 |
| **129-6** | 129, Germany (July 2012) | 1024 | 383 | 1046 | Synonymous mutations | | | Synonymous mutations | |

**Table S2e: isogenic isolates from 2 different patients originating from the same country**

| **Isolates** | **Patient ID, country and date of collection** | **TMO**  **MIC**  **(mg/L)** | **Protein length**  **(AA)** | | **Encoded MexA** | | **Encoded MexB** | |
| --- | --- | --- | --- | --- | --- | --- | --- | --- |
|  |  |  | **MexA** | **MexB** | **Side view** | **Top view** | **Side view** | **Top view** |
| **135-1** | 135, Germany (July 2012) | 8 | 383 | 719 | 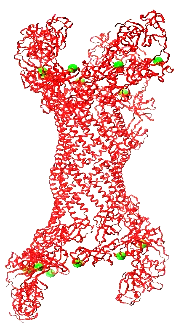 | 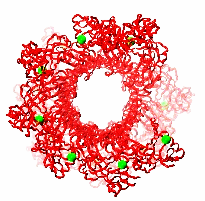 | 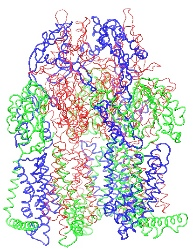 | 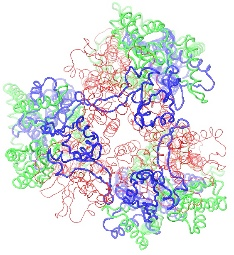 |
| **208-3** | 208, Germany (Aug. 2012) | 256 | 383 | 1045 | 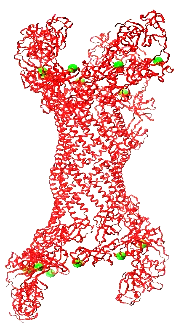 | 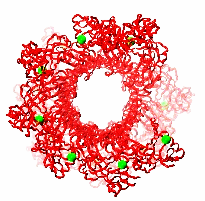 | 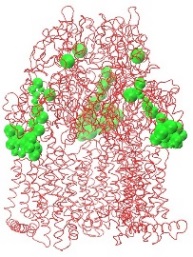 | 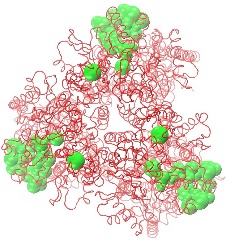 |
| **208-2** | 208, Germany (Aug. 2012) | 512 | 383 | 1045 | 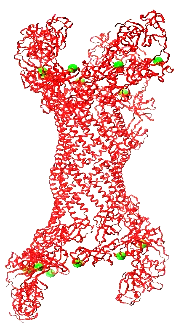 | 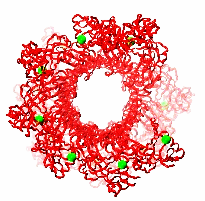 | 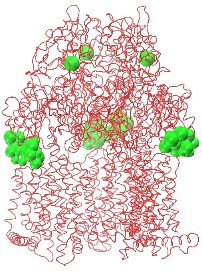 | 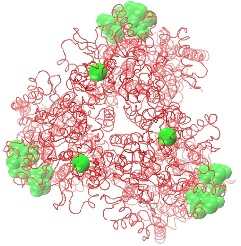 |
